# Treatment for problem gambling and counselors’ perception of their clinical competence: a national web survey in Sweden

**DOI:** 10.1186/s13722-022-00347-w

**Published:** 2022-12-09

**Authors:** Viktor Månsson, Eva Samuelsson, Anne H. Berman, Anders Nilsson

**Affiliations:** 1 Centre for Psychiatry Research, Department of Clinical Neuroscience, Karolinska Institutet, Region Stockholm, Norra Stationsgatan 69, 113 64 Stockholm, Sweden; 2 Department of Social Work, Department of Public Health Sciences, Centre for Social Research On Alcohol and Drugs (SoRAD), Stockholm University, Stockholm, Sweden; 3 Department of Psychology, Uppsala University, Uppsala, Sweden

**Keywords:** Problem gambling, Treatment, Counselors, Role security, Clinical competence

## Abstract

**Background:**

Despite their crucial role in bridging science and practice, not much is known about counselors offering treatment for Problem Gambling (PG). This study maps current treatment, the type of change techniques that are prioritized in treatment and how counselors perceive their clinical competence in their work with PG clients.

**Methods:**

A sample of PG counselors from the healthcare and social services (N = 188, mean age: 49 years, 67% women) completed an online survey. A principal component analysis was conducted to map prioritized types of change techniques, and a multiple regression analysis was carried out to analyze predictors of counselors’ role adequacy in their clinical work.

**Results:**

There was a large variation in the type of treatments offered for PG (mean 3.6). Cognitive Behavioral Therapy (CBT) and Motivational Interviewing were the most common treatments offered and motivation was rated as the most important type of change technique prioritized in the treatment of PG. A principal component analysis identified four components reflecting different types of change techniques prioritized by the counselors: (1) standard CBT, e.g., gambling cognitions, craving management, and finding alternative activities, (2) assessment of PG, (3) family orientation, i.e., involvement of concerned significant others in treatment, and (4) focus on exposure strategies. Counseling more clients monthly was associated with higher levels of willingness, adequacy and legitimacy in their clinical work with clients with PG. Additionally, offering CBT was a predictor for higher role adequacy and providing counseling on the origins of and consequences of PG.

**Conclusion:**

There was a large heterogeneity among the treatments offered and what change techniques that were prioritized among the PG counselors. Clinical experience is of importance for developing competence in treating clients with PG. This finding suggests there could be benefits to establishing specialized, more visible treatment units where PG counselors could gain adequate clinical experience, thus increasing clinical competence for treating PG.

## Introduction

Problem gambling (PG) counselors constitute the bridge between scientifically supported treatment and the client and dissemination of such treatment relies on the counselors` competence and willingness to accept the scientific foundation for their work. Despite this, little research attention has been paid to them.

Treatment for PG is scarce in many parts of the world and bridging the gap between research and practice is a major challenge. Knowledge of PG among treatment providers has been lacking, help-seeking rates are low, and research on PG has been lagging behind that on substance use [1]. Meta-analyses of treatment studies show the short-term effectiveness of Cognitive Behavioral Therapy (CBT) in reducing gambling behavior and symptoms of PG [1–3], but only a handful of studies have investigated the long-term effects. From a CBT perspective, PG is a learned behavior and focuses on raising awareness of dysfunctional behavior patterns and gambling cognitions and cravings, developing skills to prevent relapses, and promoting alternative behaviors [4, 5]. Motivational Interviewing (MI), often delivered as a brief intervention for sub-diagnostic problems, focuses on evoking change talk, as a way of strengthening inner motivation that may otherwise be weakened by a client’s ambivalence towards change [5, 6]. MI, delivered separately or in combination with CBT, has shown effectiveness in reducing money spent on gambling [6, 7]. Other psychosocial treatments for PG have insufficient scientific support and there are currently no approved pharmacological treatments for PG, due to divergent trial results [7, 8]. It should be noted that CBT encompasses a range of interventions, from exposure-based techniques to cognitive restructuring and third-wave therapies including mindfulness and values-based work to relapse prevention [9]. Indeed, CBT is often delivered as a combination of several strategies originating from learning theory.

Studies show that the counselors’ perceptions and characteristics can be important in the dissemination and quality of treatment [10]. Within the addiction field, counselor attitudes have for example been shown to be of importance for the prescription rates of naltrexone for alcohol use disorder [11] and the availability of motivational incentives in addiction treatment [12]. Working as a PG counselor involves supporting clients who might relapse [13], commonly have both health and financial problems [14], express ambivalence towards making behavior changes and often discontinue treatment prematurely [15]. Therefore, the role of the PG counselor can be experienced as multifaceted, with interventions directed both at the gambling behavior, improving other health outcomes, and at times involving concerned significant others (CSOs) in the treatment. The clinical work can thus require a high level of *role security*, meaning a sense of psychological safety towards working with the specific population and having adequate skills to carry out the clinical task [16]. The concept of role security entails *adequacy*; i.e., having enough knowledge to carry out one´s work and *legitimacy*; i.e., experiencing it as appropriate to inquire about the addictive behavior. These factors together with a *willingness* to work with the designated task and *self-esteem* in the specific task might be important for PG counselors to be effective in their clinical tasks.

The term PG, used throughout this article, refers to a continuum of negative financial, social, and health-related consequences from gambling and thus includes gambling problems at varying levels of burdens for the individual, from mild to severe gambling problems [17]. Even though not as commonly diagnosed and treated as other addictive disorders, treatment uptake for PG in Sweden is increasing. Prevalence surveys show that about 1.4% of the Swedish population report signs of PG within the past 12 months, 0.7% with more severe problems, potentially above the threshold for a diagnosis of Gambling Disorder (GD), as outlined in the Diagnostical and Statistical Manual (DSM 5) [18, 19]. Furthermore, studies conducted on Swedish patient registers from specialized healthcare show an increase in the prevalence of the GD diagnosis (F63.0 according to the ICD-system) up until 2016 (n = 324) [20], but still represents only a small fraction of individuals with PG.

Current knowledge gaps concerning PG treatment offered in Sweden include the type of change techniques used, as well as counselors’ perceived competence in delivering PG treatment. Additionally, it is not known how practicing PG counselors prioritize among available types of change techniques. The aims of this study are therefore to (1) map the treatment offered for PG in Sweden, (2) identify the types of change techniques prioritized by PG counselors, and (3) explore factors associated with perceived clinical competence in providing treatment, i.e., willingness, adequacy, legitimacy, and self-esteem in relation to working with clients with PG.

## Materials and methods

### Design and setting

In Sweden, legislation concerning the treatment of PG has been subject to substantial recent reform [21]. The organization of public responsibility for offering support and treatment for PG was stipulated in legislation enacted in 2018 [21], and the availability of gambling-specific outpatient treatment in the 290 municipal social services increased considerably from 7% in 2015 to 74% in 2019 (n = 177 out of 239 responding municipalities) [22]. National treatment recommendations were formulated and disseminated [8] and the demand for staff training in healthcare and social services increased. This cross-sectional online survey targeted all practicing PG counselors in Sweden within the public domain, including addiction units within municipal social services as well as specialized addiction services within the healthcare system. Data were collected between March 23 and April 27 in 2021 using the web-survey tool Survey-Xact [23].

### Recruitment

The study targeted all counselors who had been providing counseling for PG in the public domain during the prior 12 months. The invitation to participate was emailed to all 290 municipalities organizing social services in Sweden and the 21 regions organizing healthcare. Based on the national helpline register, it is estimated that at least 214 Swedish municipalities offered specific treatment for PG in 2019, either by employed counselors or through contracts with private companies [22]. In addition, the invitation was sent to a national network of PG counselors organized by two of the authors (VM, AN), meaning that some received the invitation twice. To avoid duplicate responses, each participant was assigned a unique ID in the survey. The more inclusive PG term was applied throughout the survey since it was assumed that not all counselors were utilizing the Swedish term for diagnosis of Gambling Disorder (*Hasardspelsyndrom*). No reimbursement was offered to participants. Recruitment lasted for 1 month and it was estimated to take 20 min to complete the survey. The questionnaire did not contain any questions regarding the name or location of the clinic or addiction center or the name or email address of the counselor to reduce the risk of social desirability effects in the responses, as well as to comply with data management stipulations in accordance with the General Data Protection Regulation (GDPR: 23) in the European Union.

### Participant characteristics

The recruitment procedure led to the inclusion of 188 PG counselors, with a large majority (n = 163) working within the social services organized by the municipalities and the remainder working within the healthcare system; i.e., psychiatric services and primary care. Among the respondents, 71.8% reported counseling fewer than two clients with PG monthly. See Table 1 for a description of the sample.

**Table 1:** Sample characteristics

Variable	Full sample (N = 188^a^)	Social services (n = 163)	Healthcare (n = 24)	
Age. Mean (range)	49.39 (27–68)	49.64 (27–68)	47.39 (31–64)	
% Women	67	65.4	78.3	
Years working with PG treatment (%)				
0–1	8.4	7.9	4.2	
2–3	45.2	46	41.7	
4–6	28.7	26.3	45.8	
7–10	9.6	9.7	8.3	
> 10	8.5	10.1	0	
Years in occupation. mean (sd)	16.0 (9.7)	16 (9.4)	15.3 (11.2)	
Average number of clients per month				
Basic education/profession	n (%)	≤ 2 clients (%)	3–10 clients (%)	> 10 clients (%)
Social worker	68 (36.2)	72.1	23.5	4.4
Behavioral scientist	24 (12.8)	79.2	16.7	4.2
Pedagogic counselor	16 (8.5)	68.8	31.3	0.0
Medical training	14 (7.4)	64.3	21.4	14.3
Alcohol and drug therapist	13 (6.9)	53.8	30.8	15.4
Assistant nurse	11 (5.9)	81.8	18.2	0.0
Psychologist	10 (5.3)	70.0	20.0	10.0
Other	31 (16.4)	74.2	25.8	0.0
Total	188 (100)	71.8	22.3	5.9

^a^The full sample included 1 counselor in private practice. Healthcare included psychiatry (n = 20) and primary care (n = 4). Medical training = medical doctors, nurses, physiotherapists and occupational therapists. Other = police, economist, youth worker or not specifying basic profession

### Materials

The survey consisted of the following five parts: (1) information regarding study participation and data management; and (2) sociodemographic questions. This was followed by (3) questions on the type of treatment offered based on a list of PG interventions stemming from systematic reviews [1, 7] and the authors’ knowledge from the field. The list of treatments can be categorized as those under the CBT umbrella and thus stemming from learning theory, CBT, *Acceptance and Commitment Therapy* (ACT) [24], *Community Reinforcement Therapy* (CRA) [25] *Relapse Prevention* [26] *Community Reinforcement Therapy and Family Training* (CRAFT) [27] those based on *Motivational Interviewing*, MI and Motivational Enhancement Therapy (MET) [28], *twelve-step facilitation* [29], i.e., adhering to the twelve steps to recovery as described by Gamblers Anonymous (GA) and non-specific supportive counseling (individual or together with CSOs). A free text “other” category was added for types of treatment not listed. After this, questions followed on (4) delivery modes (e.g., group, individual counseling, online treatment); and (5) the importance of including the following types of change techniques in the treatment; *motivation, assessment of PG, functional analysis, craving management, involving CSOs, self-exclusion from gambling, gambling cognitions, avoiding risk situations, attention to children in families with PG, psychoeducation* (on topics such as the brain’s reward system), *mindfulness practice, exposure, preventing relapses, values clarification*, and finding *alternatives to gambling*. These were rated from not at all important (= 1) to crucial (= 5), with an additional response option if the counselor lacked experience in working with the specific component. The list was based on PG treatment manuals evaluated in a meta-analysis, as well as literature on change techniques used in PG treatment [30–32].

Counselors’ attitudes and perceptions towards working with clients with PG were measured using an adapted version of the *Short Alcohol and Alcohol Problems Questionnaire* (SAAPPQ) [16]. Since the instrument has been used to measure attitudes toward working with individuals with problem drinking, the wording was changed to be directed at working with clients with PG (e.g., “I want to work with drinkers” was changed to “I want to work with clients with problem gambling”). The scale consists of 10 items and answers were scored on a 7-point Likert scale ranging from “strongly agree” to “strongly disagree”. The scale measures four factors: (1) willingness to work with clients with PG; (2) role adequacy, meaning adequacy of knowledge and skills in working with clients with PG; (3) legitimacy, meaning an experience of having the legitimacy to ask questions regarding gambling habits and (4) experiencing self-esteem in the specific task as well as work satisfaction. Adequacy and legitimacy are categorized as part of the concept of *role security*, and willingness and self-esteem are categorized under *therapeutic commitment*. A longitudinal psychometric investigation of SAAPPQ concluded that the global scale should not be used due to low correlation among the factors, but only the four factors outlined [16].

### Data preparation and statistical analysis

The data set was first screened for duplicates. The variable concerning type of treatment available was recoded as CBT when participants reported Relapse Prevention or named a specific CBT manual. The profession variable was recoded based on the number of years of higher education for a degree; e.g., 1 = less than 3 years of education or a professional status not requiring higher education, 2 = 3–5 years, e.g., social workers and nurses and 3) 5 or more years, mainly licensed psychologists and medical doctors.

A regression analysis was conducted with five variables entered as predictors: (1) years working with PG; (2) gender; (3) workplace (the healthcare system or social services); (4) the average number of monthly clients with PG; (5) offering CBT; (6) offering MI; and (7) years of professional education at the three levels described. The approach was exploratory, and no prior hypothesis was set. All predictors were entered simultaneously into the model.

To explore patterns in the responses and correlations among the types of change techniques prioritized in treatment, a Principal Component Analysis (PCA) was carried out using Varimax factor rotation. This was done to summarize the data and group the counselors by their differing prioritization of types of change techniques. The number of factors was selected following a visual inspection of the scree plot, with eigenvalues ≥ 1 aiming at including the maximum number of factors, since over-extraction is recommended as compared to under-extraction to reduce error in the model [26]. The statistical analyses were conducted using IBM SPSS Statistics (Version 26.0).

## Results

### Type of treatment offered and mode of delivery

Overall, the counselors reported offering an average of 3.6 psychosocial treatments for PG; counselors from municipalities reported 3.8 treatment types, in comparison to 2.2 treatment types among counselors in the healthcare system. The most commonly offered treatment was CBT, which was more prevalent within the healthcare system; see Table 2 for an overview of the types of treatment offered. Social services and the healthcare system differed mainly in that the social services offered Community Reinforcement Approach (CRA), Community Reinforcement Approach and Family Training (CRAFT), and Twelve-step facilitation to some extent, whereas the healthcare system did not offer any of these treatment types.

**Table 2:** Types of PG treatment offered by counselors in social services and within the healthcare system

Type of treatment	Full sample (%)	Social services (%)	Healthcare (%)
CBT	76.5	73.6	95.8
MI	74.3	77.9	50
Supportive counseling	54.6	57.7	33.3
Support for CSOs	37.2	41.1	8.3
Twelve step facilitation	35.5	41	0
CRA	28.4	33.1	0
ACT	14.2	15.3	8.3
CRAFT	13.7	16	0
Pharmacological	3.8	1.9	16.7
PDT	3.3	3.1	4.2
Other:	10.9	10.7	13.0

*CBT* cognitive behavior therapy. Pharmacological = Naltrexon and Nalmefen. *MI* Motivational Interviewing, *ACT* acceptance and commitment therapy, *CSO* Concerned Significant Other, *CRA* community reinforcement approach. *CRAFT* community reinforcement approach and family training. *PDT* psychodynamic therapy, Other: includes Problem Solving therapy. Motivational Enhancement Therapy. Previct (digital intervention)

Regarding treatment delivery modes, almost all respondents (98.4%) reported delivering treatment through individual counseling. Social services offered treatment to individuals attending together with CSOs to a larger extent (68.9%) than the healthcare system (34.8%). About one-third (35.5%) of the whole sample offered treatment through video calls and 10.4% offered internet treatment. About half of the sample (51.4%) offered group treatment.

### Prioritization of types of change techniques

**Fig. 1:**
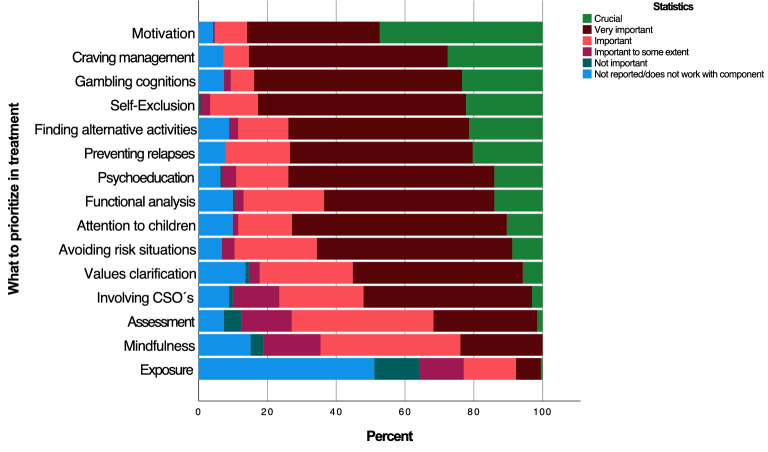
Types of change techniques, sorted from most to least prioritized

Figure 1 displays the counselors’ responses concerning what types of change techniques they prioritized in treatment. Motivational enhancement was rated as the most important change technique, followed by the CBT-techniques of craving management and gambling cognitions. Half of the sample reported not working with exposure techniques.

### Principal component analysis

The Kaiser–Meyer–Olkin Measure of Sampling Adequacy = 0.8574 and Bartlett’s Test of Sphericity were both significant (p < 0.001), suggesting data were suitable for reduction techniques such as PCA. The PCA yielded four components with eigenvalues ≥ 1, explaining 56.81% of the total variance (32.28%, 9.69%, 8.02% and 6.82%, respectively for components 1–4).

The first component, termed *Standard CBT,* explained about one-third of the variance, represented a standard CBT package delivered for PG, including such components as motivation, craving management, gambling cognitions, relapse prevention, avoiding risk situations, psychoeducation, and finding alternatives to gambling. This content mirrors that of typical CBT treatments for PG presented in available manuals. The second component, termed *Assessment and self-help,* included assessment, functional analysis, self-exclusion and mindfulness practice. This content can be viewed as representing treatment content delivered by non-experienced PG counselors, or counselors working primarily with brief interventions and self-help interventions, where the main task is to screen, assess and recommend self-exclusion. The third component, termed *Family/couple*, contained family and couple orientations, including attention to children in families with PG and involving concerned significant others in treatment. It is worth noting that motivation was not rated as important change technique among this group (loading of − 0.37). The fourth component, termed *Exposure*, represented counselors who rated exposure strategies as important. Functional analysis is typically a part of exposure-based treatment (loading 0.31) and the utilization of self-exclusion tools loaded negatively in this component (− 0.73), as self-exclusion can reduce opportunities to practice exposure. See Table 3 for an overview of component loadings.

**Table 3:** Principal Component Analysis. Rotated Component Matrix of prioritization of types of change technique

Types of change technique	Component			
	1: Standard CBT	2: Assessment and self help	3: Family/couple	4: Exposure
Gambling Cognitions	**0.82**	0.13	0.26	
Craving Management	**0.76**			
Preventing relapses	**0.76**	0.13	0.13	0.16
Avoiding risk situations	**0.73**		0.26	− 0.19
Psychoeducation	**0.67**	0.34		
Finding alternative activities	**0.57**	0.11	0.28	0.11
Motivation	**0.51**	0.37	− 0.37	0.13
Functional analysis	0.33	**0.40**	0.30	0.31
Mindfulness	0.33	**0.60**		− 0.13
Values clarification	0.27	0.33	0.32	0.27
Attention to children	0.24	0.28	**0.50**	
Involving CSOs	0.16		**0.81**	− 0.12
Exposure	0.11	0.23		**0.72**
Assessment		**0.79**	0.20	
Self-Exclusion		**0.40**		− **0.73**
% of variance explained by factor	32.28	9.69	8.02	6.82

Bold values indicate a correlation above 0.40

Extraction Method: Principal Component Analysis. a. Rotation converged in 6 iterations. Factor loadings > .40 highlighted. Correlations − 0.1 to 0.1 not displayed. Rotation Method: Varimax with Kaiser Normalization

### Willingness, role adequacy and legitimacy

Three of the four factors covered by the SAAPPQ showed satisfactory internal reliability consistency: willingness (Cronbach’s α = 0.65), adequacy (Cronbach’s α = 0.72) and legitimacy (Cronbach’s α = 0.85). The self-esteem factor showed unsatisfactory internal reliability consistency (Cronbach’s α = 0.40) and was removed from the analysis. Therefore, three multiple regression analyses were run with *willingness*, *adequacy,* and *legitimacy* as dependent variables. A significant model predicted *willingness* (R^2^ = 0.15, F (7, 184) = 4.52, p < 0.001), a significant model predicted *adequacy* (R^2^ = 0.14, F (7, 185) = 4.17, p < 0.001) and a non-significant model was found for *legitimacy* (R^2^ = 0.06, F (7, 184) = 1.80, p = 0.09).

The results of the multiple regression analyses are shown in Table 4. Seeing more clients with PG per month was positively associated with the three remaining factors, willingness, adequacy, and legitimacy, of the SAAPPQ. In addition, offering CBT was associated with higher role adequacy overall. We found no significant association between sex, workplace (healthcare/social services), years working as a PG counselor, offering MI, or level of education with any of the factors in SAAPPQ.

**Table 4:** Output from the three multiple regression analyses with the SAAPPQ subscales as dependent variables

Willingness							Adequacy						Legitimacy					
Model: Enter	B	SE	Beta	t	95.0% CI for B [LL. UL]		B	SE	Beta	t	95.0% CI for B [LL. UL]		B	SE	Beta	t	95.0% CI for B [LL. UL]	
(Constant)	10.49	0.89		11.79	8.73	12.24	10.79	0.88		12.32	9.06	12.52	12.71	0.76		16.81	11.22	14.21
Sex	0.10	0.30	0.02	0.33	− 0.50	0.70	0.15	0.30	0.03	0.50	− 0.44	0.74	0.07	0.26	0.02	0.29	− 0.43	0.58
Years PG-counselor	0.02	0.03	0.04	0.58	− 0.04	0.08	0.03	0.03	0.06	0.83	− 0.04	0.09	-0.04	0.03	− 0.11	− 1.57	− 0.09	0.01
Workplace	− 0.20	0.47	− 0.03	− 0.41	− 1.13	0.74	0.05	0.47	0.01	0.11	− 0.87	0.98	− 0.35	0.40	− 0.07	− 0.86	− 1.14	0.45
No. of PG-clients	**0.68**	0.14	**0.34**	4.75	0.40	0.96	**0.40**	0.14	**0.20**	2.82	0.12	0.67	**0.32**	0.12	**0.20**	2.66	0.08	0.56
Offers CBT	0.37	0.34	0.08	1.10	− 0.29	1.03	**1.00**	0.33	**0.21**	3.02	0.35	1.65	0.38	0.29	0.10	1.34	− 0.18	0.95
Offers MI	− 0.61	0.33	− 0.13	− 1.83	− 1.26	0.05	− 0.67	0.33	− 0.15	− 2.06	− 1.31	− 0.03	− 0.15	0.28	− 0.04	− 0.53	− 0.70	0.41
Education (3 levels)	− 0.02	0.26	− 0.01	− 0.09	− 0.53	0.49	− 0.36	0.25	− 0.11	− 1.44	− 0.87	0.14	− 0.18	0.22	− 0.06	− 0.82	− 0.61	0.25

Bold values indicate significance at *p* < 0.01

Multiple Linear regression output. All variables entered simultaneously. Bold indicates significant at p<.01. B = Unstandardized Coefficients. SE = Standard Error. Beta = Standardized Coefficients. CI = Confidence Intervals Workplace coded as 1= Municipality and 2 = Healthcare. LL = Lower Limit. UL = Upper Limit.

## Discussion

This study investigates the activities and perceptions of counselors providing PG treatment, covering the treatment content they offer, the types of change techniques they prioritize, and how they perceive their clinical competence when working with clients with PG. The results showed that CBT and MI, the two treatment types with the most robust scientific support hitherto, form the most commonly offered treatment for PG in Sweden. Overall, addressing motivation during treatment was viewed as the most important type of change technique, a factor that can be considered a prerequisite for other interventions. According to the principal components analysis, about one-third of the counselors were prioritizing “standard CBT” content in their treatment, while a minority were focusing on assessment, families, and exposure strategies. Furthermore, counseling more clients monthly was associated with higher levels of role security, adequacy, and legitimacy in carrying out their clinical tasks.

The most common prioritization of change techniques in treatment was using a range of what we termed a “standard CBT” toolkit. This mirrors the collection of strategies put forward by Rodda et al. [31] as helpful in reducing gambling behavior; after analyzing responses from 489 gamblers, Rodda et al. suggest that interventions for PG should target cognitions such as reminding oneself of negative consequences from gambling, feedback on gambling behavior, planning ahead and craving management, resembling the “standard CBT” component identified in the present study. In addition, a review of types of change techniques reported in clinical trials [32] found that relapse prevention was the most common type of change technique, utilized in 60% of studies, followed by cognitive restructuring, behavioral substitution (termed “alternative activities” in the present study) and stimulus control. All four of these techniques were rated among the six most important change techniques in the present study, preceded only by motivation and craving management.

Interestingly, the strategy of stimulus control varies over time and between countries. A majority of counselors (82.8%) in the present study rated the utilization of self-exclusion from gambling, a stimulus control strategy, as crucial or very important. This change technique refers to the national register introduced in Sweden in 2019 where citizens can self-exclude from all licensed gambling and direct commercials for a period of one, three, six, or 12 months. However, concerns have been raised that a large proportion of individuals with PG continue to gamble outside the licensed market [33, 34] and that relying on self-exclusion might impact the motivation to continue treatment and lead to premature dropout. In this way, the external nature of stimulus control such as self-exclusion tools and limiting access to money can be associated with persons with PG depriving themselves of the opportunity to gain internal stimulus control of the type provided by exposure-based strategies and relapse prevention techniques. External and internal stimulus control strategies can, optimally, function as complementary to one another. Nevertheless, access to effective self-exclusion tools—even without complementary treatment—constitutes a public health strategy that contributes to reducing the negative impact of online and land-based gambling [35].

Somewhat surprisingly, gender, age, and the number of years of clinical work with PG were not associated with any of the factors of SAAPPQ, whereas seeing more clients monthly was positively correlated with higher willingness, adequacy, and legitimacy. The direction of causality is not known but one can speculate that willingness to work with clients with PG might influence a counselor to see more clients. On the other hand, gaining experience from the assessment and treatment of clients with PG can be reflected in increased adequacy and legitimacy in the execution of one’s work. Other unmeasured factors could influence this association, such as working in an environment that promotes role security in the clinical task, a factor more likely to be present for counselors working at specialized PG units, in addition to their having the opportunity to treat PG clients regularly.

Gaining sufficient clinical experience in the field of PG can be challenging due to low rates of treatment-seeking among individuals with PG. About 5–12% of individuals with PG report seeking any formal help, suggesting that a large majority deal with their problems outside formal treatment systems [19, 36]. PG-related stigma is one of the most frequently cited barriers to seeking help [37] and mental health stigma, in general, has a wide-ranging negative impact on the lives of individuals suffering and affects the allocation of resources to healthcare, as a systematic review concludes [38]. In addition to low rates of treatment-seeking, discontinuing treatment is common; a meta-analysis reports that 39.1% of clients with PG drop out of treatment [39]. This might cause a vicious circle where counselors do not obtain sufficient experience, affecting their clinical competence and possibly also the quality of their treatment skills and the attractiveness of treatment. Repetition and deliberate practice are pivotal in developing expert skills [40] and for the development of clinical skills within medical education [41] and, in this study, for self-perceived role security in the treatment of PG.

Offering CBT was positively associated with higher perceived role adequacy in the present study. This might be at least partly due to the often-emphasized skill within CBT of psychoeducation as a part of the treatment and the availability of treatment manuals with CBT content. Nevertheless, delivering CBT for PG requires training, supervision, and an organization that supports evidence-based treatment, factors highlighted as important when implementing treatment within the addiction care [42]. More clarity in the provision of PG treatment, perhaps through specialized units, might contribute to increased role security among counselors and facilitate help-seeking through a clear path to access help.

Substantial challenges exist in transferring research and policy into clinical practice. The implementation of evidence-based methods within addiction care is dependent on the consensus of researchers, treatment administrators, clinicians, and patients, where both ethical and financial incentives are of importance [43]. The complexity among clients and multiple tasks at hand can thus present obstacles in adhering to manual-based treatment interventions.

Another difference between research and practice is the basic profession of the counselor, which is hypothesized to influence the core competence and the delivery of treatment. It should be noted that the CBT evaluated in clinical trials is commonly delivered by clinical psychologists with specific PG training and supervision [30]. Nevertheless, the current study did not find any significant differences in perceived role security between counselors with different lengths of professional training.

One strength of the current study is the proportion of counselors included, most likely representing most practicing PG counselors in Sweden at the time of data collection. A second strength is the finding that further investigation is needed regarding role security among PG counselors, where additional studies into the conceptualization of role security and the psychometric properties of the SAAPPQ are needed. Some limitations also need to be addressed. One regards the lack of a baseline measure of counselors’ characteristics and competence before the new legislation was introduced in 2018 and the impossibility of thus tracking changes in the counselors’ role security over time. Calculating the exact proportion of active counselors responding to the invitation was difficult due to changes in the workforce and a lack of information on the number of active PG counselors the previous year. However, the recruitment strategy was extensive, both through official channels and a mailing list containing those who participated in PG education.

Additionally, the possible bias in the responses due to self-report and social desirability are limitations that might slant the results towards the over-reporting of recommended treatments. However, personal data on the respondent were not collected, to counteract this risk and thereby increase the internal validity of the responses. Also worth noting is that the sample consisted of many counselors with limited clinical experience. This circumstance might be a product of the strategy of increasing the availability of treatment geographically through educating more counselors, while the number of individuals with PG seeking treatment remains low. A final remark is that the study was conducted in April 2021 when the restrictions due to the COVID-19 pandemic were in place, limiting physical contact in parallel with recommendations to work from home, if possible, which impacted the availability of on-site group treatment.

To our knowledge, this is the first study investigating PG counselors on a national level. Given the development of addiction treatment, starting in the mid-twentieth century when paraprofessionals transitioning to counselors were the most common treatment providers and moving towards increasing prevalence of healthcare professionals in the early twenty-first century [44], it is important to track this continuing professionalization and its impact on practice. The dissemination of treatment due to new legislative acts and re-organization of treatment offers an opportunity to investigate emerging practices within the helping systems.

## Conclusions

To conclude, research on the perceptions of PG counselors is underexplored. More studies are needed to investigate what constitutes an efficacious and clinically competent counselor within PG and what factors support and promote the development of such a counselor. Additionally, it would be of value to further explore the concept of role security, its relation to treatment outcome, and the occupational well-being of the counselor, particularly in comparison with counselors for other addictive behaviors.

CBT and MI are the most common treatments offered for PG in Sweden. Addressing motivation was seen as the most important change technique in treatment, and counselors seeing more clients monthly reported higher levels of willingness, adequacy, and legitimacy in their clinical work with clients with PG. This points toward the potential benefits of specialized, more visible, treatment units where PG counselors can gain adequate clinical experience.

## Data Availability

The data that support the findings of this study are available from the corresponding author, (VM) upon reasonable request.

## Abbreviations

CBT: Cognitive behavioral therapy
CRA: Community reinforcement approach
CRAFT: Community reinforcement approach and family training
CSO: Concerned significant other
MI: Motivational interviewing
PG: Problem gambling
SAAPPQ: Short alcohol and alcohol problems questionnaire

## References

[1] Cowlishaw S, Merkouris S, Dowling N, Anderson C, Jackson A, Thomas S (2012). Psychological therapies for pathological and problem gambling. Cochrane Database Syst Rev.

[2] Petry NM, Ginley MK, Rash CJ (2017). A systematic review of treatments for problem gambling. Psychol Addict Behav.

[3] Ribeiro EO, Afonso NH, Morgado P (2021). Non-pharmacological treatment of gambling disorder: a systematic review of randomized controlled trials. BMC Psychiatry.

[4] Petry NM, Ammerman Y, Bohl J, Doersch A, Gay H, Kadden R (2006). Cognitive-behavioral therapy for pathological gamblers. J Consult Clin Psychol.

[5] Rash CJ, Petry NM (2014). Psychological treatments for gambling disorder. Psychol Res Behav Manag.

[6] Yakovenko I, Quigley L, Hemmelgarn BR, Hodgins DC, Ronksley P (2015). The efficacy of motivational interviewing for disordered gambling: systematic review and meta-analysis. Addict Behav.

[7] Di Nicola M, De Crescenzo F, D’Alò GL, Remondi C, Panaccione I, Moccia L (2020). Pharmacological and psychosocial treatment of adults with gambling disorder: a meta-review. J Addict Med.

[8] National Board of Health and Welfare. Behandling av spelmissbruk och spelberoende. Kunskapsstöd med rekommendationer till hälso- och sjukvården och socialtjänsten [Treatment of gambling problems and gambling disorder. Knowledge support with recommendations for healthcare and social services.] 2018. [cited 2022Nov.21]. Available from:https://www.socialstyrelsen.se/globalassets/sharepointdokument/ artikelkatalog/kunskapsstod/2018-12-5.pdf.

[9] Tolchard B (2017). Cognitive-behavior therapy for problem gambling: a critique of current treatments and proposed new unified approach. J Ment Health.

[10] Waller G, Turner H (2015). Therapist drift redux: Why well-meaning clinicians fail to deliver evidence-based therapy, and how to get back on track. Behav Res Ther.

[11] Abraham AJ, Rieckmann T, McNulty T, Kovas AE, Roman PM (2011). Counselor attitudes toward the use of naltrexone in substance abuse treatment: a multi-level modeling approach. Addict Behav.

[12] Ducharme LJ, Knudsen HK, Abraham AJ, Roman PM (2010). Counselor attitudes toward the use of motivational incentives in addiction treatment. Am J Addict.

[13] Ledgerwood DM, Petry NM (2006). What do we know about relapse in pathological gambling?. Clin Psychol Rev.

[14] Dowling N, Merkouris SS, Lorains FK (2016). Interventions for comorbid problem gambling and psychiatric disorders: advancing a developing field of research. Addict Behav.

[15] Nilsson A, Simonsson O, Hellner C (2021). Reasons for dropping out of internet-based problem gambling treatment, and the process of recovery—a qualitative assessment. Curr Psychol..

[16] Richardson GB, Smith R, Lowe L, Acquavita SP (2020). Structure and longitudinal invariance of the short alcohol and alcohol problems perception questionnaire. J Subst Abuse Treat.

[17] Hodgins DC, Stea JN, Grant JE (2011). Gambling disorders. The Lancet.

[18] American Psychiatric Association. Diagnostic and statistical manual of mental disorders. 5th ed (text rev.). 2022. 10.1176/appi.books.9780890425787

[19] Abbott M, Romild U, Volberg R. The prevalence, incidence, and gender and age-specific incidence of problem gambling: results of the Swedish longitudinal gambling study (Swelogs). Addiction 2018;113(4):699–707. 10.1111/add.14083. 10.1111/add.14083 29105942

[20] Hakansson A, Karlsson A, Widinghoff C (2018). Primary and secondary diagnoses of gambling disorder and psychiatric comorbidity in the swedish health care system-a nationwide register study. Front Psychiatry.

[21] Prop. 2016/17:85. https://data.riksdagen.se/fil/E7C9555F-BD43-4E06-9748-EF39B8DBC02A; 2016.

[22] Berman AH. Stödlinjen. Årsrapport 2019 [Annual report of Gambling Helpline]. http://dok.slso.sll.se/CPF/Stodlinjen/Stodlinjens_arsrapport_2019.pdf; 2019 4th Jan 2021.

[23] Voigt P, Von dem Bussche A (2017). The eu general data protection regulation (gdpr) A Practical Guide.

[24] Dixon MR, Wilson AN, Habib R (2016). Neurological evidence of acceptance and commitment therapy effectiveness in college-age gamblers. J Contextual Behav Sci.

[25] Miller WR, Meyers RJ (2004). The community-reinforcement approach. Psychosocial Treatments.

[26] Marlatt GA, George WH (1984). Relapse prevention: introduction and overview of the model. Br J Addict.

[27] Meyers RJ, Miller WR, Hill DE, Tonigan JS (1998). Community reinforcement and family training (CRAFT): engaging unmotivated drug users in treatment. J Subst Abuse.

[28] Hodgins DC, Currie SR, Guebaly-el N (2001). Motivational enhancement and self-help treatments for problem gambling. J Consult Clin Psychol.

[29] Marceaux JC, Melville CL (2011). Twelve-step facilitated versus mapping-enhanced cognitive-behavioral therapy for pathological gambling: a controlled study. J Gambl Stud.

[30] Gooding P, Tarrier N (2009). A systematic review and meta-analysis of cognitive-behavioural interventions to reduce problem gambling: hedging our bets?. Behav Res Ther.

[31] Rodda SN, Bagot KL, Cheetham A, Hodgins DC, Hing N, Lubman DI (2018). Types of change strategies for limiting or reducing gambling behaviors and their perceived helpfulness: a factor analysis. Psychol Addict Behav.

[32] Rodda S, Merkouris SS, Abraham C, Hodgins DC, Cowlishaw S, Dowling NA (2018). Therapist-delivered and self-help interventions for gambling problems: a review of contents. J Behav Addict.

[33] Håkansson A, Widinghoff C (2020). Gambling despite nationwide self-exclusion-a survey in online gamblers in Sweden. Front Psychiatry.

[34] Månsson V, Wall H, Berman AH, Jayaram-Lindstrom N, Rosendahl I (2021). A longitudinal study of gambling behaviors during the COVID-19 pandemic in Sweden. Front Psychol.

[35] Gainsbury SM (2014). Review of self-exclusion from gambling venues as an intervention for problem gambling. J Gambl Stud.

[36] Slutske WS (2006). Natural recovery and treatment-seeking in pathological gambling: results of two U.S national surveys. Am J Psychiatry.

[37] Suurvali H, Cordingley J, Hodgins DC, Cunningham J (2009). Barriers to seeking help for gambling problems: a review of the empirical literature. J Gambl Stud.

[38] Sharac J, Mccrone P, Clement S, Thornicroft G (2010). The economic impact of mental health stigma and discrimination: a systematic review. Epidemiol Psychiatric Sci.

[39] Pfund RA, Peter SC, McAfee NW, Ginley MK, Whelan JP, Meyers AW (2021). Dropout from face-to-face, multi-session psychological treatments for problem and disordered gambling: a systematic review and meta-analysis. Psychol Addict Behav.

[40] Ericsson KA (2004). Deliberate practice and the acquisition and maintenance of expert performance in medicine and related domains. Acad Med.

[41] Duvivier RJ, van Dalen J, Muijtjens AM, Moulaert VR, van der Vleuten CP, Scherpbier AJ (2011). The role of deliberate practice in the acquisition of clinical skills. BMC Med Educ.

[42] Amodeo M, Lundgren L, Cohen A, Rose D, Chassler D, Beltrame C (2011). Barriers to implementing evidence-based practices in addiction treatment programs: comparing staff reports on motivational interviewing, adolescent community reinforcement approach, assertive community treatment, and cognitive-behavioral therapy. Eval Program Plann.

[43] Drake RE, Goldman HH, Leff HS, Lehman AF, Dixon L, Mueser KT (2001). Implementing evidence-based practices in routine mental health service settings. Psychiatr Serv.

[44] Mulvey KP, Hubbard S, Hayashi S (2003). A national study of the substance abuse treatment workforce. J Subst Abuse Treat.

